# Serum lactate normalization time associated with prolonged postoperative ileus after surgical management of the small bowel and/or mesenteric injuries

**DOI:** 10.1186/s12893-024-02388-1

**Published:** 2024-03-21

**Authors:** Naa Lee, Euisung Jeong, Yunchul Park, Younggoun Jo, Jungchul Kim, Hyunseok Jang

**Affiliations:** Division of Trauma, Department of Surgery, Chonnam National University Medical School and Hospital, 42 Jebong- ro, Dong-gu, Gwangju, 61469 Republic of Korea

**Keywords:** Abdominal injury, Enteral nutrition, Ileus, Lactate

## Abstract

Back ground: Determining the optimal timing of postoperative oral feeding in trauma patients who have undergone abdominal surgery with small bowel and/or mesenteric injuries is challenging. The aim of this study is to investigate serum lactate as a factor that can predict oral feeding tolerance and prolonged postoperative ileus (PPOI) in patients who underwent surgery for small bowel and/or mesenteric injury due to trauma.

Methods: The single center retrospective observational study was conducted on 367 patients who underwent surgery for small bowel and/or mesenteric injury between January 2013 and July 2021. The patient group was divided into two groups based on whether the peak serum lactate was over 2mmol/L (18 mg/dL). In the group of lactate > 2mmol/L, it was divided into prolonged postoperative ileus (PPOI) groups and groups rather than PPOI.

Results: Patients in the peak serum lactate > 2 group had tendency to use vasopressors, lower initial systolic blood pressure, larger number of packed red blood cells for 24 h, higher injury severity score, higher PPOI incidence, and a tendency for delayed oral intake tolerance. In peak serum lactate greater than 2 mmol/L group, the lactate normalization time (OR 1.699, *p* = 0.04), quantity of FFP transfusion for 24 h (OR 1.145, *p* = 0.012), and creatine kinase (OR 1.001, *p* = 0.023) were related to PPOI. The lactate normalization time had the highest correlation.

Conclusion: In patients undergoing surgical management for small bowel and/or mesenteric injury after trauma, serum lactate normalization time affects oral intake tolerance and prolongs postoperative ileus.

**Supplementary Information:**

The online version contains supplementary material available at 10.1186/s12893-024-02388-1.

## Introduction

The timing of postoperative oral feeding in patients who have undergone abdominal surgery has long been discussed. Currently, early postoperative feeding and normal food intake are strongly recommended in abdominal surgery, and the timing of oral intake, which is an essential part of Enhanced Recovery After Surgery (ERAS) protocols, has remained a subject of discussion in critically ill and emergency patients [1]. Early oral feeding and enteral nutrition are known to maintain gastrointestinal (GI) integrity, improve function, reduce infection, hospital stay, cost, and mortality, and consequently improve patient clinical outcomes [2]. According to the established guidelines, if hemodynamically stable, it is recommended to start early enteral nutrition within 24 to 48 h in trauma and critically ill patients to improve their outcomes [3–6]. Despite the strong recommendation, there are many difficulties in starting early oral feeding to trauma patients with various injuries or patients recovering from shock.

In the case of abdominal trauma, small bowel and/or mesenteric injury accounts for 1–5% and is the most common hollow viscus organ injury [7, 8]. Unlike other solid organ injuries, such as liver or spleen, traumatic small bowel and/or mesenteric injury directly involves the GI tract. Due to the involvement of the GI tract, surgeons hesitate to start oral feeding because of the risk of anastomotic leak and delayed bowel function recovery. Therefore, early oral feeding and appropriate timing of oral intake remain major concerns and are not commonly applied, even in elective surgery [9].

These factors make it difficult to determine the optimal timing of oral feeding in trauma patients undergoing emergency surgery. According to early enteral nutrition in critically ill patients’ clinical guidelines by the European Society of Intensive Care Medicine (ESICM), enteral nutrition could be delayed in patients with uncontrolled shock or bowel ischemia [4]. Even if early enteral nutrition is started, it is not easy to achieve calorie and protein goals in a short time. There are cases in which early enteral nutrition is stopped due to hemodynamic instability, gastrointestinal tract complications, and other conditions [10]. In the state of enteral nutrition intolerance, complications might worsen the patient’s physiological state [11, 12]. Complications due to enteral nutrition intolerance can cause fatal complications, especially in patients with multiple traumas. In patients who underwent surgery for small bowel and/or mesenteric injury, more studies are needed in predicting the possibility of oral intake tolerance and the timing of oral intake. In one study, enteral feeding intolerance in patients with sepsis was reported to be associated with elevated serum lactate levels [13]. Furthermore, lactate is an important indicator of shock and is highly correlated with prognosis in trauma patients. Therefore, we studied the relationship between postoperative oral feeding tolerance and lactate levels in patients who underwent operative management after small bowel and/or mesenteric injury due to trauma.

### Patients and methods

This was a retrospective study where we reviewed 367 patients who underwent surgical treatment for small bowel and/or mesenteric injury at the Chonnam National University Hospital regional trauma center, Gwangju, Korea, between January 2013 and July 2021. This study was approved by the Chonnam National University Institutional Review Board (IRB CNUH-2023-060). The requirement for informed consent was waived by the IRB.

**Fig. 1:**
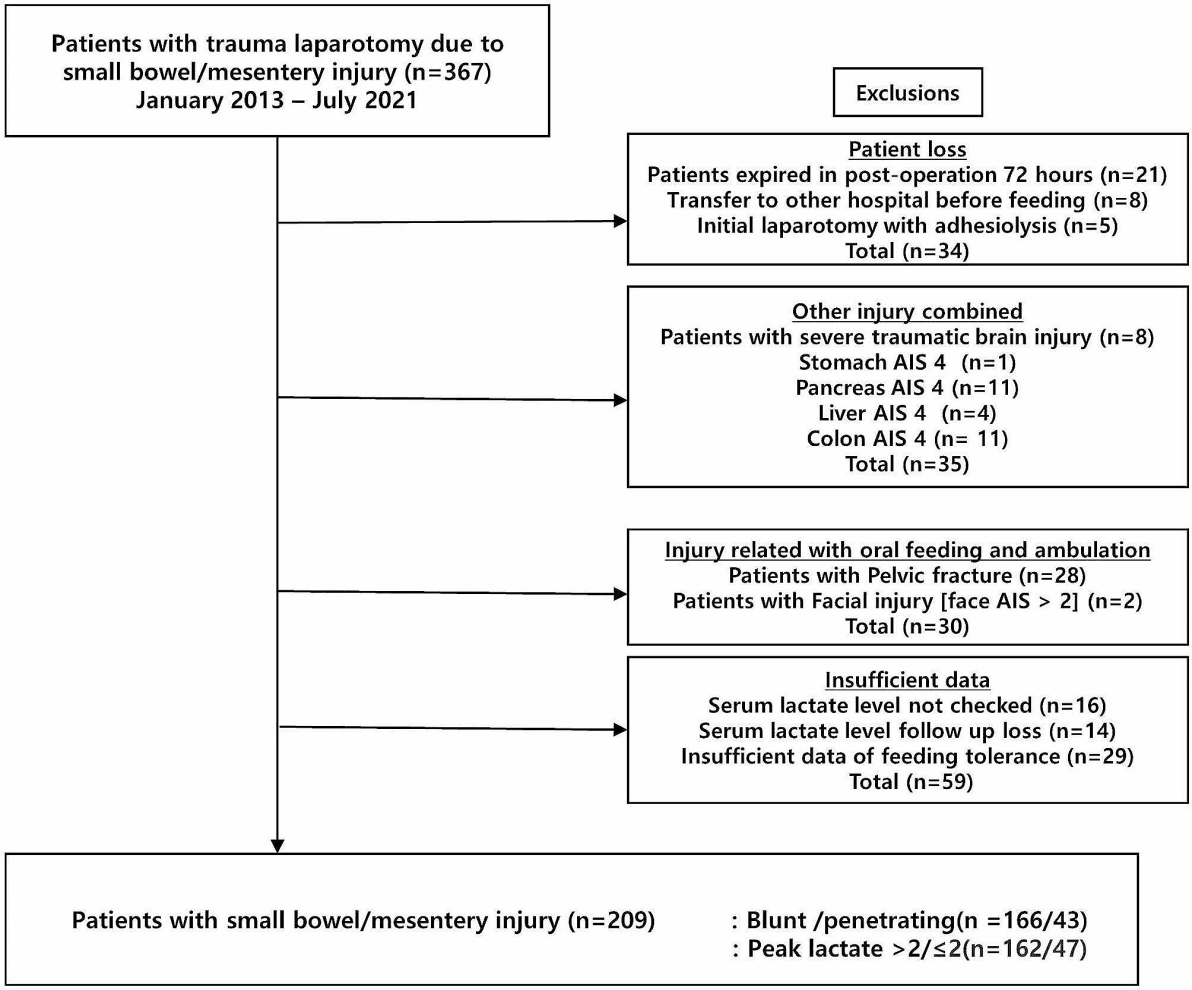
Study inclusion flowchart

As shown in Fig. 1, among the 367 patients, 34 were excluded, including 21 who died within 72 h, 8 who were transferred to another hospital before feeding initiation, and 5 who underwent adhesion-lysis with initial laparotomy due to pre-existing adhesion. Patients with other organ damage in the abdominal cavity with a 4 or higher abbreviated injury scale (AIS) score (stomach, *n* = 1; pancreas, *n* = 11; liver, *n* = 4; colon, *n* = 11) and those with severe traumatic brain injury (Glasgow Coma Scale score < 9) were excluded. Patients who had a facial injury (face AIS > 2) related to oral feeding and pelvic fractures which could affect ambulation and recovery of bowel function were excluded (facial injury, *n* = 2; pelvic injury, *n* = 28). Fifty-nine patients were excluded due to insufficient data, and 209 patients were analyzed.

For each patient group, prehospital time, initial white blood cells (WBC), hemoglobin (Hb), platelet (PLT), albumin, initial serum lactate, peak serum lactate during the hospital stay, systolic blood pressure, use of vasopressor or inotropes, cardiopulmonary resuscitation, quantity of crystalloid, packed red blood cells (pRBC), fresh frozen plasma (FFP), platelet concentrate (PC) infusion within the first 24 h, abdomen abbreviated injury scale (AIS), injury severity score (ISS) were analyzed for their relationship with the day of oral feeding tolerance and prolonged postoperative ileus. In the case of serum lactate, a level exceeding 2 mmol/L (18 mg/dL) was defined as a marker of circulatory and cellular/metabolic abnormalities according to the International Consensus Definitions for Sepsis and Septic Shock. In this study, the analysis was performed by dividing the patient group based on the peak lactate 2 mmol/L. In addition, the serum lactate normalization time was separately checked in the group with lactate exceeding 2 mmol/L, which was defined as the time required for serum lactate to decrease from greater than 2 mmol/L to less than 2 mmol/L [14]. Serum lactate levels were periodically measured throughout the patient care process, beginning with the initial assessment upon the patient’s arrival at the emergency room as part of routine laboratory tests. During surgery, lactate measurements were conducted at least once, with additional assessments at the anesthesiologist’s discretion based on the patient’s condition and the progression of the surgery. This approach allowed for real-time monitoring of lactate levels and the adjustment of clinical management as needed. Additional measurements post-surgery were determined by the physician, based on the patient’s status. These intervals ranged from 30 min to 8 h, tailored to each patient’s individual needs, particularly if there were signs of deterioration. This flexible strategy was crucial for closely monitoring each patient’s recovery and responding promptly to any changes in their condition. After surgery, patients were admitted to the ICU where routine laboratory tests, including serum lactate levels, were conducted. For those admitted to the ICU, serum lactate levels were measured daily at approximately 6 a.m. The aim of this comprehensive and dynamic approach to monitoring was to ensure effective tracking of lactate normalization, thereby guiding appropriate and timely clinical interventions.

For oral feeding, water was first given when the patient’s vital signs stabilized. To prevent aspiration pneumonia, nasogastric tubes were routinely inserted before or during emergency surgeries. These tubes were then removed as early as possible post-surgery, allowing for the initiation of oral feeding in patients who were cooperative and assessed to be at low risk of aspiration. This practice was integral to our protocol, especially for patients who could safely resume oral intake. Total Parenteral Nutrition (TPN) was administered selectively to patients who were unable to receive oral nutrition due to their critical condition. TPN was used depending on the physiological status of the patient. Solid diet was initiated unless there were new gastrointestinal (GI) tract symptoms, or evidence of ileus on radiographic examination. Prolonged postoperative ileus (PPOI) was defined as no passage of flatus or stool and no oral feeding tolerance after 4 postoperative days, or those that satisfied at least two of the five criteria (nausea or vomiting, no oral feeding tolerance in the last 24 h, no flatus in the last 24 h, abdominal distention, or radiologic ileus) [15]. Supplemental Parenteral Nutrition (SPN) was used if patients’ nutritional requirements were not met through oral feeding.

The Mann–Whitney U test was used to compare the two groups, whereas Fisher’s exact test was used to compare categorical data. A p-value < 0.05 was considered statistically significant. Simple linear, multiple linear, and logistic regression analyses were performed for PPOI. Multiple linear regression was performed by selecting only variables with a p-value of less than 0.05 in univariate analysis. Finally, the model was selected by performing stepwise backward elimination and regression of all subsets. Each statistical analysis was conducted based on R software, ver. 3.6 (R Foundation for statistical computing, Vienna, Austria).

## Results

Among the 209 patients, 166 (79.4%) had blunt injuries, and 43 had penetrating injuries. Among 209 patients, 162 (77.5%) had peak serum lactate exceeding 2 mmol/L. Table 1 shows the results of the comparison between the group with a peak lactate level of ≤ 2 mmol/L and the group with a peak lactate > 2 mmol/L. In addition to the differences in initial serum lactate and peak serum lactate levels between the two groups, there was a higher tendency to use vasopressors and lower initial systolic blood pressure in the group with peak lactate > 2 mmol/L. In addition, the quantity of number and pRBC infusion for 24 h was higher in the lactate > 2 group. In the case of AIS, there was no significant difference between the two groups; however, patients with a lactate > 2 mmol/L had a higher risk of ISS. In addition, patients in the lactate > 2 group had a higher PPOI and a tendency for delayed oral intake tolerance.

**Table 1:** Patient demographics

	Peak lactate ≤ 2		Peak lactate > 2		p	
	(*N* = 47)		(*N* = 162)			
Age	52.7 ± 16.9		52.4 ± 16.4		0.916	
Sex					0.381	
- F	14 (29.8%)		36 (22.2%)			
- M	33 (70.2%)		126 (77.8%)			
Time taken to visit ER (min)	721.3 ± 1892.6		259.5 ± 557.8		0.105	
Initial WBC (10^3^/uL)	12.0 ± 6.0		13.7 ± 6.4		0.091	
Initial Hb (g/dL)	12.4 ± 2.1		12.5 ± 2.5		0.736	
Initial PLT (10^3^/uL)	227.7 ± 75.1		211.4 ± 63.6		0.139	
Albumin (g/dL)	3.5 ± 0.5		3.4 ± 0.7		0.611	
Creatine Kinase (IU/L)	375.9 ± 433.7		549.0 ± 885.0		0.072	
Initial lactate (mmol/L)	1.2 ± 0.4		4.2 ± 3.3		**< 0.001**	
Peak lactate (mmol/L)	1.2 ± 0.4		5.3 ± 3.3		**< 0.001**	
Inotropics and vasopressor use					**0.018**	
not use	42 (89.4%)		115 (71.0%)			
use	5 (10.6%)		47 (29.0%)			
Initial sBP (mmHg)	107.9 ± 21.6		98.8 ± 24.2		**0.022**	
Crystalloid (mL)	2126.6 ± 1206.0		3066.4 ± 1622.3		**< 0.001**	
pRBC (unit)	2.6 ± 4.0		4.1 ± 5.2		**0.036**	
FFP (unit)	2.1 ± 3.1		3.1 ± 4.2		0.058	
PC (unit)	1.5 ± 4.7		2.3 ± 4.7		0.338	
AIS of abdomen					0.657	
2	3 ( 6.4%)		12 ( 7.4%)			
3	29 (61.7%)		87 (53.7%)			
4	15 (31.9%)		60 (37.0%)			
5	0 ( 0.0%)		3 ( 1.9%)			
AIS of small bowel					0.827	
0	15 (31.9%)		58 (35.8%)			
2	2 ( 4.3%)		6 ( 3.7%)			
3	25 (53.2%)		75 (46.3%)			
4	5 (10.6%)		23 (14.2%)			
AIS of mesentery					0.065	
0	23 (48.9%)		49 (30.2%)			
2	5 (10.6%)		35 (21.6%)			
3	11 (23.4%)		36 (22.2%)			
4	8 (17.0%)		42 (25.9%)			
ISS	12.7 ± 5.1		15.7 ± 7.1		**0.002**	
Oral feeding tolerance day	5.2 ± 3.6		6.8 ± 4.7		**0.019**	
PPOI					**0.012**	
-	28 (59.6%)		61 (37.7%)			
+	19 (40.4%)		101 (62.3%)			

Categorical variables are expressed as numbers (%), and the continuous variables are presented as medians [first and third quartiles]

WBC, white blood cells; Hb,hemoglobin; PLT, platelet; sBP, systolic blood pressure; pRBC, packed red blood cell; FFP, fresh frozen plasma; PC platelet concentrate; AIS, Abbreviated Injury Scale; ISS, Injury Severity Score; PPOI prolonged postoperative ileus

There were differences between the groups with PPOI and non-PPOI, with peak lactate levels greater than 2 mmol/L. The non-PPOI patients had higher initial Hb, platelet, and albumin levels; lower creatine kinase, initial lactate, and peak lactate levels; shorter time to lactate normalization; tended to use inotropics and vasopressors; and lower amounts of crystalloid, pRBC, FFP, and PC transfusions for the initial 24 h. Furthermore, small bowel AIS was higher in the PPOI group, and ISS was also higher. Oral feeding tolerance occurred earlier in the non-PPOI group (Table 2).

**Table 2:** Comparison between non-PPOI and PPOI group in peak lactate greater than 2 mmol/L patients

	non-PPOI	PPOI	p
	(*N* = 61)	(*N* = 101)	
Age	49.2 ± 16.1	54.2 ± 16.4	0.06
Sex			0.862
- F	14 (23.0%)	22 (21.8%)	
- M	47 (77.0%)	79 (78.2%)	
Time taken to visit ER (min)	197.9 ± 212.6	296.8 ± 685.7	0.978
BMI (kg/m^2^)	23.9 ± 3.2	24.2 ± 3.2	0.561
Initial WBC (10^3^/uL)	14.7 ± 6.9	13.2 ± 6.0	0.159
Initial Hb (g/dL)	13.0 ± 2.4	12.2 ± 2.6	**0.044**
Initial PLT (10^3^/uL)	223.7 ± 45.9	203.9 ± 71.4	**0.033**
Albumin (g/dL)	3.6 ± 0.7	3.3 ± 0.7	**0.014**
Creatine kinase (IU/L)	294.9 ± 297.3	698.9 ± 1066.3	**0.043**
Initial lactate (mmol/L)	3.7 ± 3.3	4.5 ± 3.4	**0.026**
Peak lactate (mmol/L)	4.7 ± 3.1	5.7 ± 3.4	**0.012**
Lactate normalization time (day)	1.2 ± 0.6	1.6 ± 0.9	**0.001**
Inotropics and vasopressor			**0.042**
not use	49 (80.3%)	66 (65.3%)	
use	12 (19.7%)	35 (34.7%)	
Initial sBP (mmHg)	101.8 ± 20.7	97.0 ± 26.0	0.222
Crystalloid (mL)	2665.6 ± 1329.4	3308.4 ± 1737.7	**0.009**
pRBC (unit)	2.6 ± 3.4	5.0 ± 5.9	**0.001**
FFP (unit)	1.9 ± 2.5	3.9 ± 4.8	**0.001**
PC (unit)	1.2 ± 3.6	2.9 ± 5.2	**0.015**
AIS of abdomen			0.052
2	5 ( 8.2%)	7 ( 6.9%)	
3	40 (65.6%)	47 (46.5%)	
4	16 (26.2%)	44 (43.6%)	
5	0 ( 0.0%)	3 ( 3.0%)	
AIS of small bowel			**0.005**
0	24 (39.3%)	34 (33.7%)	
2	1 ( 1.6%)	5 ( 5.0%)	
3	34 (55.7%)	41 (40.6%)	
4	2 ( 3.3%)	21 (20.8%)	
AIS of mesentery			0.716
0	18 (29.5%)	31 (30.7%)	
2	16 (26.2%)	19 (18.8%)	
3	13 (21.3%)	23 (22.8%)	
4	14 (23.0%)	28 (27.7%)	
ISS	13.6 ± 6.4	17.0 ± 7.3	**0.003**
Oral feeding tolerance day	3.3 ± 0.9	8.9 ± 4.7	**< 0.001**

Categorical variables are expressed as numbers (%), and the continuous variables are presented as medians [first and third quartiles]

BMI, body mass index ;WBC, white blood cells; Hb,hemoglobin; PLT, platelet; sBP, systolic blood pressure; pRBC, packed red blood cell; FFP, fresh frozen plasma; PC platelet concentrate; AIS, Abbreviated Injury Scale; ISS, Injury Severity Score

The PPOI-related factor was analyzed by multiple regression analysis and a model using data from the group with a peak lactate greater than 2 mmol/L group. In the final model, the lactate normalization time (OR 1.699, *p* = 0.04), quantity of FFP transfusion for 24 h (OR 1.145, *p* = 0.012), and CK (OR 1.001, *p* = 0.023) were related to PPOI. The lactate normalization time had the highest correlation (Table 3).

**Table 3:** Related factor of PPOI in peak lactate greater than 2 mmol/L group in multiple regression analysis and finally selected model

	univariate analysis		multivariate analysis		final selected model	
	OR(95% CI)	p	OR(95% CI)	p	OR(95% CI)	p
Age	1.019 (0.999–1.039)	0.062				
Initial Hb	0.874 (0.765–0.998)	0.046	0.926 (0.751–1.143)	0.474		
Initial PLT	0.995 (0.990 -1.0002)	0.059				
Albumin	0.551 (0.339–0.895)	0.016	1.037 (0.474–2.268)	0.928		
Creatine kinase	1.001 (1.0002–1.002)	0.02	1.001 (1.00009–1.002)	0.032	**1.001** **(1.0001–1.002)**	**0.023**
Initial lactate	1.078 (0.965–1.205)	0.183				
Peak lactate	1.110 (0.988–1.248)	0.08				
Lactate normalization time	2.042 (1.254–3.325)	0.004	1.773 (1.019–3.084)	0.043	**1.699** **(1.024–2.821)**	**0.04**
Inotropics and vasopressor	2.165 (1.020–4.596)	0.044	1.330 (0.542–3.265)	0.534		
Initial systolic BP	0.992 (0.978–1.005)	0.222				
Crystalloid	1.0003 (1.00005–1.0005)	0.016	0.9999 (0.9996–1.0003)	0.757		
pRBC	1.113 (1.030–1.202)	0.007				
FFP	1.153 (1.044–1.274)	0.005	1.137 (0.948–1.363)	0.166	**1.145** **(1.031–1.273)**	**0.012**
PC	1.097 (1.007–1.195)	0.035	0.966 (0.857–1.090)	0.578		
AIS of abdomen	1.864 (1.104–3.146)	0.02	1.527 (0.688–3.392)	0.298		
AIS of small bowel	1.135 (0.927–1.389)	0.221				
ISS	1.076 (1.024–1.131)	0.004	0.987 (0.912–1.068)	0.744		

Hb,hemoglobin; PLT, platelet; pRBC, packed red blood cell; FFP, fresh frozen plasma; PC platelet concentrate; AIS, Abbreviated Injury Scale; ISS, Injury Severity Score

## Discussion

Many studies have shown that early enteral nutrition after gastrointestinal surgery can help reduce the length of hospital stay, infectious complications, and mortality [16]. Early enteral nutrition should be considered for patients who can tolerate it. However, most trauma patients are in a state of physiological derangement and cannot tolerate enteral feeding. In the context of physiological derangement states, there are various causes and mechanisms of oral feeding intolerance. It is known to be related to GI tract stretching and injury, fluid overload, opioids, and neurohormonal dysfunction [11, 12]. . In addition, trauma patients are accompanied by organ injuries as well as surrounding muscles, and require a multimodal approach and intensive care [17]. For this reason, it is difficult to start oral feeding by simply considering the recovery of bowel function. In addition, when patients experience trauma, they are in a state of ischemia-reperfusion and immune inflammatory response. Studies on the process of multiorgan failure through this mechanism are being actively conducted [18]. The initiation time of oral feeding should be carefully considered.

However, few studies have focused on oral intake tolerance in patients who have undergone abdominal surgery and traumatic injury. According to the ESICM clinical guidelines, delayed enteral nutrition is recommended in patients with uncontrolled shock or bowel ischemia [4]. There was a previous study that reported that early enteral nutrition in mechanically ventilated patients can cause pneumonia, diarrhea, and an increase in the length of hospital stay [19]. Moreover, in trauma patients, if trauma is related to the gastrointestinal tract, trauma-induced gastrointestinal dysfunction is a common morbidity. The gut microbiome that changes after trauma can affect gut homeostasis and barrier function [20]. Changes in GI motility and GI dysfunction were common in the group of patients who underwent surgical management for small bowel and/or mesenteric injury [21]. Considering this, the advantages of early oral feeding are clear, but the application of early oral feeding in trauma patients who often suffer from multiple injuries and shock is limited. In addition, since oral feeding intolerance (abdominal discomfort, distention, vomiting, diarrhea, etc.) in trauma patients can worsen, studies on indicators of when to start oral feeding in a group of patients who underwent surgical management for small bowel and/or mesenteric injury are needed.

In our study, the decision to delay the initiation of Total Parenteral Nutrition (TPN) was informed by recent evidence suggesting potential drawbacks of early TPN administration in critically ill patients. Studies, including those by Hermans et al. (2013), indicate that late initiation of TPN could substantially reduce the incidence of weakness and facilitate faster recovery [22]. This approach aligns with current best practices prioritizing early oral or enteral feeding, which is believed to support better clinical outcomes in trauma patients by preserving muscle function and integrity. Our strategy emphasizes the importance of individualized nutritional support, balancing the need for adequate nutrition with the potential risks associated with early TPN use in critically ill patients.

Recently, studies have been conducted to predict the timing of tolerance to oral and enteral feeding. In one study, enteral feeding intolerance in patients with sepsis was reported to be related to elevated serum lactate [13]. Serum lactate is a product of cellular metabolism that accumulates when cells undergo anaerobic metabolism. Serum lactate can reflect the patient’s tissue oxygenation state, and lactate clearance serves as a guide for resuscitation in patients with sepsis [23, 24]. In trauma patients, serum lactate and serum lactate normalization time are important indicators of patient morbidity and mortality [25, 26]. Lactate clearance is proportional to central venous oxygenation, is related to hypoperfusion and hypoxemia in shock patients, and also affects intestinal hypoperfusion. Considering this, serum lactate level and serum lactate normalization time are expected to affect oral feeding intolerance; however, to date, there have been no studies on patients who underwent surgical management for small bowel and/or mesenteric injury due to trauma. In this study, there was a difference in oral intake intolerance days and PPOI between the group with a peak lactate level ≤ 2 mmol/L and the group with a peak lactate greater than 2 mmol/L. For patients with lactate levels > 2 mmol/L, the ISS tended to be higher, which suggests that the patients tended to have multiple traumas. Moreover, in those with lactate > 2 mmol/L, serum lactate normalization time showed a stronger relationship with PPOI than peak serum lactate. The ability to clear lactate to normal levels is an important factor for bowel function recovery. Additionally, in all patients, lactate normalization time was positively correlated with peak serum lactate level (Supplement Table 1).

Furthermore, we observed that the timing of resuscitation did not significantly correlate with lactate normalization and prolonged postoperative ileus (PPOI) in patients with bowel injuries. This finding indicates that the immediate initiation of resuscitation may not have had a substantial impact on overall morbidity in this specific patient group. This outcome can be partially attributed to the study’s specific concentration on patients with bowel injuries, which might partly explain why the start of resuscitation did not show a substantial effect on lactate normalization and PPOI outcomes. While early resuscitation is generally considered crucial in trauma care, its specific impact in cases of bowel injury remains an area for further exploration. This aspect of our study suggests that more research is needed to understand the nuances of postoperative recovery in different trauma scenarios, particularly in patients with severe shock or critical conditions due to massive bleeding. A further research and analysis of these specific patient groups is essential to enhance our understanding of the impact that resuscitation timing has on postoperative outcomes. Such an investigation could lead to more customized and effective strategies in the management of trauma care.

In our study, 10 patients underwent reoperation after their initial surgery, highlighting important aspects of postoperative care. These reoperations occurred after the patients had resumed normal dietary intake and were in the process of recovery. Among them, 6 required surgery for wound incisional hernias, which took place more than 10 days postoperatively. The other 4, who had returned to normal diets and were discharged, later developed mechanical obstructions due to adhesions and underwent reoperations during the long-term follow-up period, ranging from 1 to 6 months after discharge. While normal feeding was resumed and initial feeding tolerance times were included in our study, we did not find a significant relationship between lactate level changes or lactate clearance and the occurrence of these delayed reoperations or complications. This observation highlights a limitation in our study: the relationship between lactate levels and delayed postoperative complications remains unclear, indicating a need for more research into this area and other potential contributing factors.

The limitation of this study was that it is a retrospective single-center study, and there was no assessment of whether the patients met their energy needs or the amount of oral feeding. Moreover, the use of analgesics might affect bowel function recovery but were not included in the analysis. Our research indicated a strong association between delayed serum lactate normalization time and the occurrence of PPOI. However, when considering the pathophysiology of PPOI, there may be other factors that need to be evaluated. In addition, as a tertiary referral center, we receive patients from a wide geographic area, which leads to a broad range in the time taken for patients to visit the ER. This variability contributes to the wide standard deviations observed in our data. Furthermore, patients with less severe injuries often receive initial assessments at other hospitals before being transferred to our center. This process can add to the variability in transport times. It’s also important to note that our trauma center was officially opened in 2016, and the inclusion of data from before the official opening may have further contributed to these deviations. The diversity in the severity of injuries and the evolving nature of our trauma center’s catchment area are factors that have likely influenced these variances.

Another limitation is related to the exclusion of certain patients. Specifically, patients who experienced complications related to early phase feeding were part of the 59 individuals categorized under the ‘insufficient data’ group and were consequently excluded from the study. This exclusion, while aligned with the study’s focus, represents a limitation as it may have provided additional insights into the relationship between early feeding complications and postoperative outcomes. Acknowledging this, we emphasize the complexity of postoperative recovery and the importance of ongoing research to enhance our understanding of long-term outcomes in trauma patients.

In this study, when considering oral feeding in patients who underwent surgical management after small bowel and/or mesenteric injury, the possibility of oral feeding intolerance and PPOI can be estimated through serum lactate normalization time. Additional studies are needed, but it is meaningful in that it is possible to estimate the progress of PPOI in patients.

## Conclusion

In patients undergoing surgical management for small bowel and/or mesenteric injury after trauma, serum lactate normalization time affects oral intake tolerance and prolongs postoperative ileus.

### Electronic supplementary material

Below is the link to the electronic supplementary material.

Supplementary Material 1

## Data Availability

The data that support the findings of this study are available on request from the corresponding author. The data are not publicly available due to privacy or ethical restrictions.

## Abbreviations

AIS: Abbreviated injury scale
FFP: Fresh frozen plasma
GI: Gastrointestinal
ISS: Injury severity score
PC: Platelet concentrate
PPOI: Prolonged postoperative ileus
pRBC: Packed red blood cells
