# Supplementary material for: Serum lactate normalization time associated with prolonged postoperative ileus after surgical management of the small bowel and/or mesenteric injuries
Source: BMC Surg. 2024 Mar 21;24:94. doi: 10.1186/s12893-024-02388-1 (PMC10956389; doi:10.1186/s12893-024-02388-1)
Supplement: Supplementary file 1 — Supplementary Material 1 [file 12893_2024_2388_MOESM1_ESM.docx]

Supplement table 1. Related factor of lactate normalization time in multiple regression analysis and finally selected model.

|  | multivariate analysis | | | | | | final selected model | | | | | | |
| --- | --- | --- | --- | --- | --- | --- | --- | --- | --- | --- | --- | --- | --- |
|  | std.Beta | lwr | upr | SE | t value | p |  | std.Beta | lwr | upr | SE | t value | p |
| Age | 0.06 | -0.07 | 0.20 | 0.07 | 0.939 | .349 |  |  |  |  |  |  |  |
| Initial PLT | -0.14 | -0.28 | 0.00 | 0.07 | -1.947 | .053 |  | **-0.14** | **-0.25** | **-0.02** | **0.06** | **-2.341** | **.020** |
| Albumin | 0.13 | -0.03 | 0.30 | 0.08 | 1.582 | .115 |  |  |  |  |  |  |  |
| CK | 0.01 | -0.13 | 0.15 | 0.07 | 0.143 | .886 |  |  |  |  |  |  |  |
| Initial lactate | 0.01 | -0.24 | 0.26 | 0.13 | 0.086 | .932 |  |  |  |  |  |  |  |
| Peak lactate | 0.42 | 0.18 | 0.67 | 0.12 | 3.427 | < .001 |  | **0.45** | **0.33** | **0.57** | **0.06** | **7.248** | **< .001** |
| Inotropics and vasopressor | 0.05 | -0.08 | 0.19 | 0.07 | 0.805 | .422 |  |  |  |  |  |  |  |
| Crystalloid | 0.18 | 0.01 | 0.35 | 0.09 | 2.045 | .042 |  | 0.17 | 0.05 | 0.29 | 0.06 | 2.773 | .006 |
| pRBC | -0.01 | -0.35 | 0.33 | 0.17 | -0.055 | .956 |  |  |  |  |  |  |  |
| FFP | 0.01 | -0.31 | 0.32 | 0.16 | 0.034 | .973 |  |  |  |  |  |  |  |
| PC | 0.02 | -0.16 | 0.20 | 0.09 | 0.217 | .828 |  |  |  |  |  |  |  |
| AIS of abdomen | 0.06 | -0.11 | 0.22 | 0.09 | 0.652 | .515 |  |  |  |  |  |  |  |
| AIS of small bowel | -0.04 | -0.17 | 0.10 | 0.07 | -0.523 | .602 |  |  |  |  |  |  |  |
| ISS | 0.05 | -0.13 | 0.23 | 0.09 | 0.510 | .611 |  |  |  |  |  |  |  |
|  |  |  |  |  |  |  |  |  |  |  |  |  |  |

std.Beta, standardized beta coefficient; lwr, upr, denote the lower and upper bounds of the 95% confidence intervals for these coefficients

Supplement table 2. Related factor of PPOI in multiple regression analysis and finally selected model.

|  | multivariate analysis | | | |  | final selected model | | | |
| --- | --- | --- | --- | --- | --- | --- | --- | --- | --- |
|  | OR | lcl | ucl | p |  | OR | lcl | ucl | p |
| Age | 1.01 | 0.99 | 1.03 | .328 |  | 1.01 | 1.00 | 1.03 | .117 |
| Initial Hb | 1.00 | 0.83 | 1.21 | .977 |  |  |  |  |  |
| Initial PLT | 1.00 | 0.99 | 1.00 | .483 |  |  |  |  |  |
| Albumin | 0.93 | 0.44 | 1.96 | .856 |  |  |  |  |  |
| CK | 1.00 | 1.00 | 1.00 | .053 |  | 1.00 | 1.00 | 1.00 | .024 |
| Initial lactate | 0.89 | 0.69 | 1.11 | .340 |  |  |  |  |  |
| Peak lactate | 1.18 | 0.97 | 1.51 | .139 |  | 1.09 | 0.99 | 1.21 | .115 |
| Inotropics and vasopressor | 1.23 | 0.55 | 2.81 | .612 |  |  |  |  |  |
| Crystalloid | 1.00 | 1.00 | 1.00 | .904 |  |  |  |  |  |
| pRBC | 1.03 | 0.85 | 1.24 | .787 |  |  |  |  |  |
| FFP | 1.09 | 0.88 | 1.36 | .441 |  | 1.11 | 1.02 | 1.22 | .027 |
| PC | 0.97 | 0.88 | 1.08 | .594 |  |  |  |  |  |
| AIS of abdomen | 1.36 | 0.68 | 2.75 | .387 |  | 1.48 | 0.87 | 2.52 | .147 |
| ISS | 1.01 | 0.94 | 1.08 | .831 |  |  |  |  |  |
|  |  |  |  |  |  |  |  |  |  |

lwr, upr, denote the lower and upper bounds of the 95% confidence intervals for these coefficients
